# Alpha-Fetoprotein as a Biomarker in Pregnancy: From Genetic Disorders to Obstetric Complications

**DOI:** 10.3390/cimb48050534

**Published:** 2026-05-20

**Authors:** Shaqraa Musawi

**Affiliations:** Department of Medical Laboratory Technology, College of Nursing and Health Sciences, Jazan University, Jazan 45142, Saudi Arabia; smusawi@jazanu.edu.sa

**Keywords:** alpha-fetoprotein, biomarker, fetal anomalies, cancer, pregnancy complications, prenatal screening

## Abstract

Alpha-fetoprotein (AFP) is a glycoprotein primarily produced by the fetal liver and yolk sac during development. It is a multifaceted biomarker with significant applications in the prenatal screening of congenital abnormalities, cancer, and other disorders. The level of AFP in maternal blood may indicate several obstetric concerns and complications during pregnancy. Atypical AFP levels are commonly utilized as a biomarker for detecting fetal anomalies, placental complications, and other pregnancy-related issues. These findings raise concerns regarding the effectiveness of screening maternal serum alpha-fetoprotein (MS-AFP) as a primary indicator of pregnancy problems and underscore the need for further investigation into the functional role of AFP throughout pregnancy. The measurement of MS-AFP has been utilized for the past four decades. It is anticipated that MS-AFP measurement will continue to be utilized as a component of integrated or sequential tests for chromosomal abnormalities and may serve as a prognostic indicator for adverse obstetric outcomes. Critically, whether AFP functions solely as a passive marker or plays active biological roles in pregnancy physiology and pathology remains unresolved, necessitating additional mechanistic investigation and discourse. This review consolidates critical data from numerous studies on AFP, focusing specifically on its diagnostic and prognostic applications for congenital abnormalities and problems during pregnancy. This review also identifies key research gaps regarding the functional biology of AFP, particularly whether AFP functions as a passive biomarker or an active participant in the pathophysiology of adverse pregnancy outcomes.

## 1. Introduction

Alpha-fetoprotein (AFP) is a significant protein that is found exclusively in mammalian embryos and is involved in both ontogenic and oncogenic growth. Bergstrand and Czar discovered AFP in human fetal sera in 1956 [1]. It functions as a carrier for several substances, including bilirubin, fatty acids, and certain medications [2,3]. AFP levels in maternal serum (MS-AFP) and amniotic fluid can assist in early detection and genetic counseling for families with congenital abnormalities. By calculating the multiple of the median (MoM) and comparing it with established reference ranges, conditions associated with fetal malformations and adverse outcomes can be identified and risk-stratified [4]. Based on the concentration of AFP in biological fluids, elevated MS-AFP and amniotic fluid AFP levels typically indicate anatomical abnormalities, such as neural tube defects (NTDs), anencephaly, ventral wall defects, gastrointestinal atresia, renal anomalies, and disruption of placental barriers [5,6]. On the other end of the spectrum, low AFP levels are associated with chromosomal abnormalities (aneuploidy), such as trisomies, as well as fetal loss, renal pyelectasis, and fetal growth restriction [7,8,9,10].

The causes of elevated MS-AFP levels in different fetal abnormalities, particularly those affecting the central nervous system and gastrointestinal tract, are mostly unclear. Similarly, the mechanisms underlying reduced MS-AFP levels in aneuploidies such as Down syndrome (trisomy 21) remains poorly understood. Although AFP serves different roles throughout fetal development, an increasing number of studies have examined AFP and its application as a tumor-specific biomarker in the past decade. Increased AFP levels are commonly observed in adults during liver carcinogenesis and various tumor processes [11,12,13]. AFP has been used as a biomarker for detecting hepatocellular cancer.

A fundamental conceptual question runs through all AFP applications: does AFP function merely as a passive biochemical marker reflecting underlying pathology, or does it actively participate in the pathophysiological mechanisms with which it is associated? This distinction between AFP as a witness and AFP as a participant has profound implications for research priorities and clinical interpretation and is addressed across multiple sections of this review.

This review aims to present a comprehensive overview of AFP, including its physiology, detection methods, biological significance during pregnancy and beyond, and its usefulness as a marker for fetal anomalies, obstetrical problems, and pregnancy complications. This review also discusses advancements in AFP screening, combined biomarker approaches, AFP screening in the era of non-invasive prenatal testing (NIPT), evolving guidelines, and the current clinical context, and future research directions.

## 2. Characteristics and Physiology of AFP

The AFP gene is located on the long arm of chromosome 4 (4q11-q13) and has two independent enhancer and silencer regions [14]. Mammalian AFP is a member of the albuminoid protein family, which is composed of three cysteine-rich domains. This family has four members: albumin (ALB), vitamin D-binding protein, AFP, and α-ALB. The close chromosomal proximity of the *AFP* and *ALB* genes (both located on chromosome 4q11-q13) reflects their evolutionary relationship as members of the albuminoid protein family. This genomic organization has important implications for gene regulation, as AFP expression enhancers active during fetal development are redirected postnatally to sustain albumin gene transcription [15,16,17]. AFP is the dominant serum protein in embryonic life as early as the first month, reflecting its critical involvement in early human development. The AFP protein consists of 609 amino acids and is a single-polypeptide chain with a molecular weight of 69 kDa and a carbohydrate content of 3–5%. AFP is a negatively charged protein and has an isoelectric point of pH 4.57 [18,19]. It is characterized by three distinct domains, referred to as domains I, II, and III, which are arranged in a triplicate pattern. These domains are formed by loops within the molecule, which are determined by the presence of disulfide bridges. As a result, the protein takes on a helical shape that resembles either a V or a U shape when examined by electron dot maps [20]. The conservation of these domains in humans, rats, mice, and bovines suggests that AFP plays a similar functional role throughout these species [21]. AFP is present in three major isoforms that vary in binding affinity for the lectin Lens culinaris agglutinin. The isoforms AFP-L1, AFP-L2, and AFP-L3 are present in variable quantities, depending on the specific clinical circumstances [22,23].

Sites of synthesis. AFP is progressively synthesized at three anatomical locations throughout fetal development. The yolk sac serves as the principal source of circulating AFP throughout the initial 4 weeks of embryonic development. Around the sixth week of gestation, the fetal liver emerges as the primary source of AFP production, with hepatocytes being responsible for most AFP synthesis during the second and third trimesters. The fetal gastrointestinal tract accounts for a minor but quantifiable proportion of AFP production throughout the second trimester. This progressive developmental transition—yolk sac → fetal liver → gastrointestinal system—explains the typical gestational fluctuations in AFP concentrations throughout the three compartments (fetal serum, amniotic fluid, and maternal serum) [1,18].

Binding partners and their biological significance. AFP is a versatile carrier protein with an extensive range of ligands. It binds to long-chain polyunsaturated fatty acids (such as arachidonic and linoleic acids), bilirubin, steroid hormones (including estrogens, androgens, and progesterone), retinoic acid, and heavy metals (copper and zinc). It also binds to certain pharmaceuticals, including warfarin and carbamazepine. The fatty acid-binding domain of the AFP molecule is located in Domain III, which is structurally comparable to the corresponding domain of albumin, indicating their common evolutionary ancestry. These binding interactions have direct biological relevance: AFP may safeguard the developing female fetus against masculinization during the crucial period of sexual differentiation by sequestering estrogens from the fetal circulation. Additionally, AFP may enhance lipid delivery to rapidly developing neural tissue during peak neurogenesis by binding and transporting long-chain fatty acids. Furthermore, AFP may confer a degree of protection against metal-mediated oxidative stress in the developing embryo by chelating heavy metals. AFP is currently being investigated as a potential therapeutic for multiple autoimmune diseases. Collectively, these actions indicate that AFP serves as an active biological agent in fetal homeostasis rather than a passive serum protein [24,25,26,27].

## 3. AFP Detection Methods: Clinical Requirements and Technological Advances

AFP was initially quantified using immunoelectrophoresis; however, this technique proved insufficiently sensitive. An innovative quantitative automated chemiluminescent enzyme immunoassay was developed for clinical use, effectively replacing and improving prior clinical assays [28,29]. In addition, advancements in AFP detection have recently led to considerable improvements in terms of sensitivity, specificity, and practicality. The clinical utility of AFP measurement fundamentally depends on the characteristics of the assay performance. For maternal serum screening during pregnancy, AFP assays must reliably detect concentrations in the range of 5–300 ng/mL (the second trimester reference range), with sufficient precision to distinguish between normal and elevated values (typically defined as >2.5 MoM). Traditional immunoassays meet these requirements for routine prenatal screening. However, several clinical scenarios require enhanced sensitivity beyond that of standard chemiluminescent immunoassay (CLIA) platforms: (1) earlier gestational detection when AFP concentrations are lower and (2) point-of-care applications in resource-limited settings, where smartphone-based technology reduces infrastructure requirements.

The most important considerations for clinical AFP assays include the following: (1) adequate sensitivity for the intended application, (2) cost-effectiveness for population-level screening programs, (3) accessibility in diverse healthcare settings, including those with limited laboratory infrastructure, (4) precision and reproducibility to minimize false-positive and false-negative results, and (5) appropriate turnaround time for clinical decision-making. Although the novel detection methods described below offer remarkable sensitivity and technical innovation, most of them exceed the requirements for routine prenatal AFP screening. Their primary value lies in specialized applications, such as early detection and monitoring of minimal residual disease, as well as in potential future applications in resource-limited settings, where simplified portable technologies could expand access to prenatal screening.

Table 1 summarizes the full technical specifications of each platform for reference. AFP detection technologies can be organized into three clinically relevant tiers. First, CLIA-based standard clinical assays meet the practical requirements for routine prenatal screening (detection range: 5–300 ng/mL, sufficient precision to distinguish values above the 2.5 MoM threshold) and remain the platform of choice for population-level MS-AFP measurement. Second, high-sensitivity platforms—including electrochemical aptasensors, surface-enhanced Raman spectroscopy (SERS), photoelectrochemical (PEC) biosensors, and enhanced fluorescence ELISA (FELISA)—achieve femtomolar to sub-picogram detection limits and are primarily valuable in oncology (early hepatocellular carcinoma detection and minimal residual disease monitoring) rather than in prenatal screening. Third, point-of-care innovations, notably smartphone-integrated digital image colorimetry using gold nanoparticles (detection limit: 0.083 ng/mL, accuracy: ~91%), offer portability and low infrastructure requirements suitable for resource-limited settings. While all three tiers are represented in the literature, clinicians rely on Tier 1 assays when interpreting prenatal AFP results; the sensitivities of Tiers 2 and 3 substantially exceed the requirements of routine prenatal screening [25].

## 4. AFP During Pregnancy and Beyond

The yolk sac and fetal hepatocytes are the sources of AFP synthesis [1,18,42]. AFP is introduced into the mother’s bloodstream during pregnancy by the placental syncytiotrophoblast cell layer. AFP can enter the maternal circulation through a non-specific process known as “spillover” [43]. This process occurs primarily through passive diffusion across the placental barrier, with AFP molecules passing through the syncytiotrophoblast layer into maternal blood following concentration gradients from fetal circulation (where concentrations are highest). The rate of spillover increases proportionally with gestational age as the placental surface area expands and permeability increases, although the placental barrier maintains selective transport that results in MS-AFP concentrations of approximately 1/100th of fetal serum levels. The synthesis of AFP has been investigated in many in vitro systems, such as rat hepatomas and isolated fetal hepatocytes [44,45]. AFP is produced during the G-1 and S stages of the cell cycle [46].

During pregnancy, fetal plasma AFP is excreted into the amniotic fluid and passes into the urine. The concentration of AFP in amniotic fluid is approximately 1/150th to 1/200th of the concentration found in fetal plasma. Additionally, the concentration of AFP in maternal serum is approximately 1/100th of the concentration observed in the amniotic fluid [47]. The standard clinical threshold for elevated MS-AFP is ≥2.5 MoM.

The highest concentrations of 3 mg/mL (3,000,000 ng/mL) in fetal serum are reached around the third month at the end of the first trimester of pregnancy (between 10 and 13 weeks) and gradually decrease as the time of birth approaches to approximately 20,000 ng/mL [43,48]. Amniotic fluid has comparable patterns of AFP concentrations but at lower levels. However, MS-AFP levels are relatively low during early pregnancy, measuring approximately 5 ng/mL at 10 weeks of pregnancy. These levels gradually increase, reaching a peak of approximately 200–300 ng/mL at 30–32 weeks, before progressively declining until delivery. Interestingly, very small amounts of AFP can be detected in non-pregnant women due to its rapid decline, typically between 0.5 and 15 ng/mL (equivalent to 0.5–15 μg/L) [49,50,51,52]. The delayed MS-AFP peak (30–32 weeks) relative to the fetal serum peak (10–13 weeks) reflects the dynamics of transplacental transfer and maternal–fetal concentration gradients. This temporal offset occurs because of the following: (1) ongoing placental transfer: even as fetal AFP production declines after the first trimester, continuous spillover from the still-elevated fetal compartment accumulates in maternal circulation; (2) increasing placental surface area: placental growth throughout the second trimester enhances the total surface area available for AFP diffusion, partially offsetting declining fetal production; (3) slower maternal clearance: AFP clearance from maternal circulation is slower than from the fetal compartment because of differences in metabolism and volume of distribution; and (4) concentration gradient effects: the large fetal-to-maternal concentration gradient (fetal levels are 100-fold higher) drives continued transfer even as absolute fetal production decreases. Consequently, MS-AFP levels rise throughout the second trimester as accumulation exceeds clearance, peaking at 30–32 weeks before declining toward term as both fetal production and placental transfer diminish [49,50,51,52].

Interestingly, the high concentrations of AFP in fetal serum (up to 3 mg/mL) suggest functional importance beyond that of a simple marker protein, as evolution would be unlikely to maintain such high expression levels for a protein without a selective advantage.

### 4.1. Nonpathological Factors Affecting the Levels of MS-AFP

**Figure 1:**
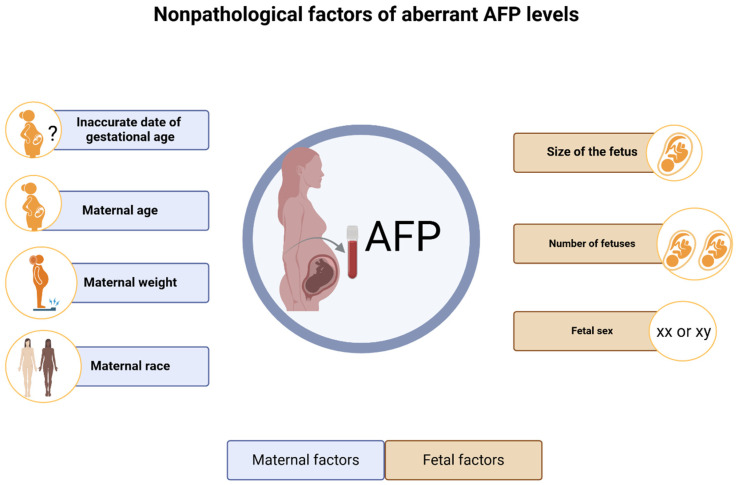
Overview of nonpathological factors affecting maternal serum alpha-fetoprotein (MS-AFP) levels. Created in BioRender. Mm, S. (2026). BioRender.com/t4jxftg.

Several physiological and procedural factors that can substantially impact MS-AFP concentrations have been investigated. Notably, nonpathological elevation of AFP can occur under several conditions. Additionally, elevated MS-AFP is associated with adverse pregnancy outcomes, including low birth weight and preterm birth, although the causal relationships and mechanisms remain under investigation. In addition, inaccurate gestational age dating—for example, when the pregnancy is more advanced than initially estimated—may result in AFP levels that are higher than expected for the assigned gestational week. Indeed, in multiple pregnancies (twins, triplets, etc.), AFP levels are approximately twice those found in singleton pregnancies, as there is an increased amount of fetal tissue producing AFP [53,54,55]. Interestingly, higher maternal age and male fetal sex are correlated with elevated MS-AFP levels compared with female fetuses, although routine sex-based adjustments are not universally applied in clinical laboratories [55,56]. In contrast, maternal weight is inversely correlated with MS-AFP levels because heavier pregnant women have lower median values due to increased blood volume [57,58]. A prospective study demonstrated an inverse relationship between glycosylated hemoglobin and MS-AFP concentrations during pregnancy in pregnant women with diabetes [59]. All these conditions must be considered when interpreting AFP levels (Figure 1).

### 4.2. Race, Ethnicity, and MS-AFP: Current Evidence and Controversy

Historically, screening programs from the early to the mid-1990s indicated modest differences in second-trimester MS-AFP based on race and ethnicity. Black women exhibited approximately 10–15% higher AFP levels than White women. Some Asian groups also demonstrated higher median levels than White and Hispanic groups, with variations observed based on gestational age. Therefore, laboratories incorporated race-specific correction factors into the MoM (e.g., increasing the AFP MoM by ~10–15% for Black women). These corrections primarily influence results close to the NTD or Down syndrome risk thresholds; results that are distinctly abnormal or distinctly normal remain unaffected [58,60].

Recent studies indicate that AFP levels in Black pregnant individuals are approximately 6–11% higher than those in White individuals at equivalent gestational ages, with race-specific median corrections reducing the screen-positive rate disparity (0.74% vs. 1.00%, *p* = 0.14) that would otherwise emerge from race-agnostic analysis [61,62]. However, another study recommended discontinuing the use of race as a factor in maternal serum screening [63]. If laboratories do not consider race, Black women may have higher false-positive screenings results and later invasive tests. If they do, they add a socially defined category to the risk assessment, which could hide underlying structural factors and reinforce the idea that biological racial differences are fixed. Nevertheless, not all laboratories consider race; some now support or have used race-neutral medians or regression models that only consider gestational age and the mother’s weight [61,64]. Finally, this issue regarding race is related not only to the AFP levels but also to other medical conditions [65]. Practical recommendation for laboratories: Given the current evidence, laboratories should apply a minimum standard of gestational age and maternal weight adjustments. Race-based correction may be considered in populations where significant AFP median differences persist after weight adjustment but should be reviewed locally and re-evaluated as demographic data accumulate. Laboratories should be transparent with clinicians about which correction model is in use, as this directly affects screen-positive rates.

After birth, the regulatory mechanisms that govern AFP change. AFP enhancers, which stimulate AFP gene transcription during fetal development, are usually suppressed from the gene promoter after birth. Instead, these enhancers function to sustain albumin gene transcription throughout adulthood [66]. In healthy term neonates, it is common to observe high blood AFP levels of up to 200,000 ng/mL during the first days of life. Then, AFP levels decrease dramatically. At the end of the first month of life, the concentrations range from 500 to 6500 ng/mL and gradually fall to levels ranging from 1 to 100 ng/mL by the age of 6 months. Subsequently, a continued and progressive decline is observed. A negligible amount of AFP can be detected in normal adult human serum, typically between 0.5 and 15 ng/mL (equivalent to 0.5–15 μg/L) [52].

## 5. AFP as a Marker for Fetal Anomalies

MS-AFP levels can function as indicators of fetal anomalies, such as NTDs and Down syndrome [67]. In the mid-1970s, NTDs were the initial developmental problems linked to aberrant AFP levels in the second trimester. AFP plays a key role in the diagnosis of fetal open NTDs, which are characterized by cerebrospinal fluid leakage into the amniotic fluid. This leakage results in absorption into the maternal bloodstream, leading to elevated MS-AFP levels [68,69,70]. The reported sensitivity of MS-AFP in detecting NTDs is approximately 95% for anencephaly and 65–80% for open spinal NTDs [71,72,73,74]. However, evidence suggests that AFP levels during early pregnancy do not specifically indicate NTDs and are more likely to reflect fetal circulation dynamics than pathological leakage [75]. Furthermore, a study demonstrated that during the second trimester, there was a notable increase in the maternal blood concentration of MS-AFP in pulmonary dysplasia-affected newborns [76]. Increased MS-AFP levels can also suggest the presence of additional fetal abnormalities, such as ventral abdominal wall defects, intestinal atresias, and sacrococcygeal teratomas. Leakage of AFP from these defective organs into the amniotic fluid leads to a higher MS-AFP concentration [72,77].

Indeed, AFP can be elevated due to the presence of certain tumors in the fetus; therefore, AFP is a useful biomarker for diagnosing, managing, and following up on select pediatric cancers, with overexpression in germ cell tumors (GCTs), hepatoblastoma (HB), and hepatocellular carcinoma (HCC) [78]. AFP is frequently increased in germ cell cancers, such as endodermal sinus tumors, which may manifest in the fetus [79]. Notably, a possible association between AFP levels and embryonic lung masses was documented in a case where AFP was increased in a fetus with Type III cystic adenomatoid lung malformation [80]. Increased AFP levels in children correlate with ovarian masses; nevertheless, further investigation is required to validate this correlation and establish its efficacy as a biomarker for ovarian mass detection [81]. Moreover, Wilms tumor is an uncommon form of kidney cancer in children. Elevated AFP levels have been observed in some patients and may be associated with tumor size and metastasis [82]. In addition, hepatic disorders, particularly HB and HCC, are associated with elevated AFP levels, particularly in children [78]. In general, in placental tumors, elevated MS-AFP levels are generally caused by their release into both the amniotic fluid and maternal placental circulation [70].

In contrast, chromosomal aneuploidies are associated with low AFP levels—for example, Down syndrome. A reduced AFP level may also be associated with Turner syndrome (45,X) and Edwards syndrome (trisomy 18), with lower sensitivity compared to Down syndrome [10,83,84]. Indeed, a recent study showed that MS-AFP could be monitored in the second trimester to detect congenital heart defects (CHD). Interestingly, AFP levels were significantly lower in mothers of neonates with CHD [85]. Additionally, a correlation was discovered between low AFP levels during the second trimester and the occurrence of sudden infant death syndrome (SIDS) in the future. The researchers in this study proposed that the risk of SIDS could be influenced, at least in part, by compromised fetal development and the occurrence of unfavorable preterm delivery events [86].

## 6. AFP as a Marker for Obstetrical Problems and Pregnancy Complications

### 6.1. From Statistical Association to Clinical Action: When Does AFP Change Management

While many AFP associations are well-cited throughout this review [5,76,87,88], a fundamental question guides clinical practice: which abnormal AFP findings actually change management and improve patient outcomes? The following distinctions are clinically relevant. [Established association] Elevated MS-AFP ≥2.5 MoM prompts Level II ultrasound and specialist referral; very high MS-AFP (≥5.0 MoM) triggers evaluation for morbidly adherent placenta (MAP) in multiparous women; and low MS-AFP (<0.7 MoM) initiate aneuploidy risk counseling and consideration of NIPT or invasive testing. Associations without proven management impact include: elevated AFP as a predictor of preterm birth, where no randomized evidence supports AFP-guided intervention, and elevated AFP in preeclampsia risk, where AFP is used in combination models but does not independently alter clinical pathways. Contexts in which AFP adds unique value beyond ultrasound include: detection of closed or skin-covered spinal defects potentially missed on ultrasound and resource-limited settings where AFP provides accessible NTD and aneuploidy risk stratification without advanced imaging infrastructure. The complete summary of clinical outcomes associated with abnormal MS-AFP levels is presented in Table 2.

### 6.2. Elevated MS-AFP: Placental Complications and Adverse Pregnancy Outcomes

Elevated MS-AFP (≥2.5 MoM) is associated with a broad spectrum of placental and obstetric complications beyond structural fetal anomalies. A prospective epidemiological study of 23,792 singleton pregnancies demonstrated that MS-AFP >2.5 MoM was associated with a higher risk of pathological pregnancies and increased risk of fetal death [89].

MAP, also referred to as the placenta accreta spectrum, is among the most clinically significant conditions associated with markedly elevated MS-AFP levels. A cohort study of 236,714 singleton pregnancies undergoing first- and second-trimester prenatal screening—excluding aneuploidies, NTDs, and abdominal wall defects—demonstrated elevated MAP risk among multiparous women with high MS-AFP [90]. A recent study further showed that MS-AFP levels exceeding 200 ng/mL in early pregnancy serve as an indicator for MAP development in later pregnancy, with a stronger predictive value than that for placenta previa (PP) [91].

Placental abruption risk has also been associated with elevated first- and second-trimester MS-AFP levels, although the predictive value for individual patients remains limited [92]. In the event of placental necrosis, disruption of the uteroplacental barrier increases AFP transfer from the fetal to the maternal compartment. A possible connection between markedly elevated serum AFP and placental necrosis was described in a case report, highlighting this as a rare but clinically significant cause of unexplained AFP elevation [93].

[Knowledge gap] A study based on pregnancy and delivery data from 5520 women between 1999 and 2014 at the University Hospital of Zurich demonstrated that pregnant women with high MS-AFP in second-trimester screening and no sonographically identified fetal abnormalities should undergo third-trimester ultrasound screening to rule out other potential pregnancy complications [94]. However, while this surveillance practice is widely recommended, there is no definitive evidence that enhanced third-trimester monitoring improves perinatal outcomes in this population. The relevant study documents the practice recommendation but does not demonstrate improvement in outcomes. [Unproven assumption].

### 6.3. Elevated MS-AFP Levels and Preterm Birth

[Established association] AFP has an established association with the prediction of preterm birth [95]. Increased AFP levels, particularly in early pregnancy, are correlated with a greater chance of preterm birth, potentially attributable to placental impairment or other underlying problems that may induce early labor. A systematic review analyzed 24 studies published between January 1991 and October 2007, evaluated 207,135 women to evaluate the association between elevated second-trimester MS-AFP and singleton preterm birth [96]. [Knowledge gap] However, a critical evidence gap exists: while third-trimester ultrasound surveillance is routinely recommended for pregnancies with unexplained elevated MS-AFP, definitive evidence that this enhanced monitoring improves perinatal outcomes is lacking. The relevant study documents the practice recommendation but does not demonstrate improvement in perinatal outcomes [94]. Similarly, the knowledge that elevated AFP predicts increased risk of preterm birth raises the practical question: how should this information guide clinical management? Current interventions for preventing preterm birth in women with isolated elevated AFP (without identified structural anomalies or placental abnormalities) are limited. Enhanced surveillance with serial growth ultrasonography, cervical length monitoring, and patient education about preterm labor symptoms are commonly implemented, but prospective randomized trials demonstrating that such interventions reduce adverse outcomes in this population are absent. [Unproven assumption] This represents a crucial area for future research: establishing whether AFP-based risk stratification leads to actionable clinical interventions that improve maternal and neonatal outcomes or whether AFP elevation primarily functions as a prognostic marker without effective therapeutic implications.

### 6.4. Elevated MS-AFP: Preeclampsia and Intrauterine Growth Restriction

Preeclampsia is a hypertensive condition of pregnancy that poses significant risks to both the mother’s and the baby’s health. Research indicates that elevated AFP levels (exceeding 2.0 MoM) are associated with an increased risk of preeclampsia, especially in early-onset cases occurring before 34 weeks of gestation [6,77]. AFP has also been evaluated in combination with other biomarkers for disease severity. Notably, AFP levels were not significantly different between the preeclampsia and control groups [97]. Moreover, intrauterine growth restriction (IUGR) is a serious disorder that restricts fetal development, increasing the risk of morbidity and mortality. Infants with IUGR encounter considerable health risks, such as increased susceptibility to metabolic syndrome, cardiovascular disease, and neurodevelopmental disorders. Timely detection of IUGR via efficient screening and monitoring can aid in managing and reducing the negative consequences linked to the condition [98]. A high AFP level of >2.0 MoM correlates with a heightened risk of IUGR. The threshold has a high specificity (94%) for predicting IUGR; however, the sensitivity is very low, suggesting that although increased AFP is a robust signal, it may not identify all instances of IUGR [99].

### 6.5. AFP:PAPP-A Ratio and Placental Insufficiency

**Figure 2:**
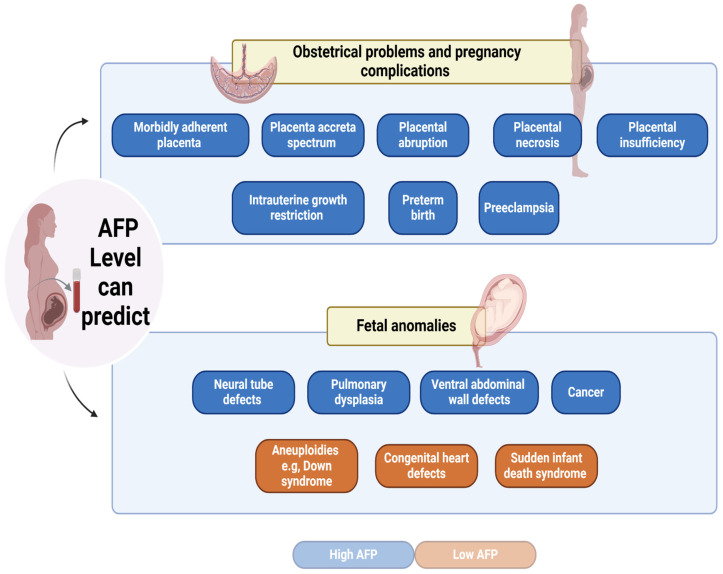
Clinical associations of abnormal maternal serum alpha-fetoprotein (MS-AFP), obstetrical problems, pregnancy complications, and fetal anomalies. Created in BioRender. Mm, S. (2026). BioRender.com/89lcad0.

A combined assessment of MS-AFP and pregnancy-associated plasma protein A (PAPP-A) provides additional predictive value for adverse placentally related outcomes beyond either marker alone. A high MS-AFP:PAPP-A ratio of >10 in the first trimester predicts placental insufficiency—a condition in which the placenta inadequately supplies nutrition and oxygen to the fetus and is associated with a range of adverse pregnancy outcomes [100]. Whether elevated AFP in pregnancy complications represents purely a marker of placental damage or contributes to pathogenesis through biological activities (immune modulation, oxidative stress, and inflammatory effects) remains an important unresolved question (Figure 2).

**Table 2:** Clinical outcomes associated with abnormal MS-AFP levels.

Condition Category	Specific Condition	AFP Level/MoM Threshold	Clinical Significance and Key Notes/References
Neural Tube Defects	Anencephaly	Elevated >2.5	High detection rate; cerebrospinal fluid (CSF) leakage from open cranial defect into amniotic fluid results in elevated MS-AFP levels [71,73]
	Open spina bifida	Elevated >2.5	Moderate sensitivity; CSF leakage through spinal defect [71,73]
	Closed spinal defects	Normal to mildly elevated	Possibly detected by AFP before visible on ultrasound [101]
Chromosomal Abnormalities	Down syndrome	Decreased <0.7	Low sensitivity alone; best used in combination with other markers [72,77,83]
	Edwards syndrome	Decreased <0.7	Sensitivity is lower than Down syndrome [72,77,83]
	Turner syndrome	Decreased <0.7	Limited screening utility as an isolated marker [83]
Congenital Heart Defects	Congenital heart defects (CHD)	Decreased <1.0	AFP levels are significantly lower in mothers of neonates with CHD; the mechanism is unclear [85]
	Sudden infant death syndrome (SIDS): future risk	Decreased <0.75	Second-trimester low AFP associated with future SIDS risk [86]
Structural Anomalies	Ventral abdominal wall defects (Gastroschisis/Omphalocele)	Elevated >2.5	AFP leakage from exposed abdominal organs; ultrasound is the primary detection [72,77,102]
	Intestinal atresias	Elevated >2.5	Ultrasound and AFP for detection [72,77]
	Sacrococcygeal teratoma	Elevated >2.5	Rare condition; AFP elevation depends on tumor characteristics [72,77]
Placental Disorders	Morbidly adherent placenta (MAP)	Elevated >2.0–2.5	Particularly in multiparous women with placenta previa [90,91]
	Placenta previa	Elevated >2.0	Overlapping presentation with MAP [91]
	Placental abruption	Elevated >2.0–2.5	Low sensitivity but significant risk elevation [92]
	Placental necrosis	Elevated >5.0	Rare condition [93]
Hypertensive Disorders	Preeclampsia (all types)	Elevated >2.0	Moderate sensitivity for overall preeclampsia [6,77]
	Early-onset preeclampsia (<34 weeks)	Elevated >2.0	Better prediction when combined with first-trimester markers [6,77,103]
Fetal Growth	Intrauterine growth restriction (IUGR)	Elevated >2.0	High specificity, low sensitivity; robust signal when present [99]
Preterm Birth	Spontaneous preterm birth	Elevated >2.0	Especially when other markers (hCG, uE3) are also abnormal [95,96]
	Very preterm birth (<32 weeks)	Elevated >2.5	Potentially attributable to placental impairment or underlying problems inducing early labor [95,96]
Placental Insufficiency	High AFP: PAPP-A ratio	Elevated AFP: PAPP-A ratio >10 (first trimester)	First-trimester ratio predictive of adverse placental outcomes [100]

## 7. Advancements in AFP Screening and Combined Biomarker Approaches

The diagnosis and management of fetal abnormalities and pregnancy problems have been greatly improved by advances in AFP screening and integrated biomarker methods. First- and second-trimester screening programs were developed with the primary goal of identifying conditions including Down syndrome and structural fetal anomalies, enabling earlier clinical decision-making. These screenings contribute to early detection, allowing for more effective care and decision-making during pregnancy [104]. Combined screening approaches including AFP, unconjugated estriol (uE3), human chorionic gonadotropin (hCG), inhibin A, and maternal age yield an 80% detection rate for Down syndrome with a 5% false-positive rate [105].

Moreover, quadruple screening (AFP, uE3, hCG, and inhibin A) during the second trimester of pregnancy can provide early indications of unfavorable maternal and fetal pregnancy outcomes [106]. The sensitivity of prenatal screening for fetal abnormalities can reach 97.93% by combining 4D color ultrasound with maternal serological tests (such as AFP, hCG, PAPP-A, and VitB12) [103]. AFP, along with first-trimester biomarkers and mean arterial pressure, is among the most effective predictors of early-onset preeclampsia [107]. A significant correlation was observed between MS-AFP levels and preterm birth when other aberrant pregnancy markers (e.g., hCG and uE3) are also raised [96]. Furthermore, unfavorable pregnancy outcomes such as preterm birth, IUGR, and macrosomia can be predicted using the first- and second-trimester serum markers PAPP-A, AFP, and maternal weight [108]. Placenta accreta spectrum (PAS) defines atypical invasion of trophoblastic tissues. AFP, along with β-hCG, PAPP-A, and cell-free fetal DNA (cffDNA), can predict PAS during pregnancy [109]. Indeed, pregnancies characterized by unexplained mid-trimester elevations in maternal serum hCG and MS-AFP are associated with an increased risk of complications due to placental insufficiency [110]. Increased AFP and hCG levels, coupled with reduced estriol, are linked to negative outcomes, including pregnancy-induced hypertension, miscarriage, preterm delivery, and intrauterine fetal death [111,112]. These methods enhance the accuracy and reliability of prenatal screening, resulting in better outcomes for both mothers and fetuses.

## 8. Evolving Guidelines and the Current Clinical Context

In recent years, the clinical value of MS-AFP screening for fetal abnormalities has been substantially re-evaluated, particularly with advances in prenatal imaging technology and the introduction of cell-free DNA (cffDNA) screening [113,114,115]. Although the American College of Obstetricians and Gynecologists (ACOG) recommended in 2003 that all pregnant women undergo MS-AFP screening during the second trimester [116], more recent guidance has shifted toward offering AFP screening as an option rather than a universal recommendation [117,118]. This transition reflects a significant change in the prenatal diagnostic field. The current scenario for AFP screening in clinical practice exhibits considerable variability and persistent debate. A study investigating prenatal screening practices across various US institutions revealed significant discrepancies in the utilization of AFP. Some centers provided it routinely, others only upon specific request, and some did not offer it at all. This variation indicates actual clinical uncertainty regarding the current value of the test [119]. Significant disparities in screening policies exist among European countries. These factors, along with organizational and cultural factors, are linked to significant variations in prenatal detection rates [102].

High-resolution ultrasound, especially when performed by skilled practitioners in tertiary centers, has shown remarkable sensitivity in identifying significant structural anomalies, including neural tube lesions. This raises the following question: Does MS-AFP offer meaningful additional diagnostic value when advanced imaging is available? One study conducted a critical evaluation of the role of MS-AFP screening in contemporary prenatal care This study examined 190,656 singleton pregnancies and found that the inclusion of MS-AFP did not significantly enhance the detection rate for spina bifida when a comprehensive second-trimester ultrasound was performed (*p* = 0.09). Ultrasound alone identified 88.6% of cases with spina bifida, whereas the combination of ultrasound and MS-AFP detected 94.3%, supporting the second-trimester MS-AFP screening methodology along with sonographic assessment in some cases [67]. In another condition, such as omphalocele, a study showed that MS-AFP screening was not associated with the early identification of an omphalocele. Moreover, in pregnancies with prenatally diagnosed omphalocele, the results of MS-AFP screening do not predict clinical outcomes [120].

Nevertheless, important points should be taken into account. Ultrasound sensitivity is significantly influenced by the operator and exhibits considerable variation across different institutions and geographic regions [121]. Second, specific spinal defects, particularly closed or skin-covered lesions, may be more effectively identified through elevated AFP levels than through ultrasound imaging [101].

The clinical utility of MS-AFP screening should be assessed in relation to healthcare resource availability. In high-resource settings where detailed anatomic ultrasound is available through maternal–fetal medicine specialists or skilled sonographers, the necessity of routine AFP screening for the detection of structural anomalies is becoming increasingly debated [67,122]. Numerous European countries, such as the Netherlands, have predominantly shifted from biochemical marker-based anomaly screening to comprehensive ultrasound examinations [123]. In resource-limited settings with restricted access to high-quality ultrasound, MS-AFP screening may still hold considerable clinical value. AFP testing requires only a blood sample and can be conducted with relatively basic laboratory facilities, making it more accessible than advanced imaging in numerous global health settings. A cost-effectiveness analysis indicates that in populations with restricted access to ultrasound, MS-AFP screening is an effective method for detecting pregnancies at heightened risk for NTDs and other anomalies [124].

Imaging techniques can detect placental abnormalities, such as previa or abruption, after their onset; however, elevated AFP levels may serve as an early biochemical indicator of placental dysfunction or compromise. The clinical applicability of this information is still under debate. MS-AFP and ultrasound are now seen as complementary tools rather than competitors in second-trimester care.

## 9. AFP Screening in the NIPT Era

NIPT, implemented in clinical practice in 2011, provides enhanced sensitivity and specificity for prevalent aneuploidies relative to traditional serum screening methods [125]. NIPT for Down syndrome attains detection rates surpassing 99% and false-positive rates under 0.1%, whereas quad screen detection rates range from 80% to 85% with false-positive rates of 5%. In addition, NIPT has high sensitivity and specificity in low-risk pregnant women [126]. In this context, is there a need for MS-AFP in contemporary practice, given that NIPT can be offered to pregnant women with a suspected fetal anomaly following an ultrasound scan?

NIPT identifies a restricted spectrum of genetic anomalies, including trisomies, sex chromosomal variations, and single microdeletions [127,128]. It should not be suggested autonomously without ultrasound evaluations that exclude malformations, growth problems, conditions characteristic of twin pregnancies, or placental pathologies [129]. Moreover, professional societies recommend that NIPT be accompanied by genetic counseling so that families can make informed reproductive choices. This raises additional challenges for NIPT uptake in developing countries with insufficient healthcare professionals and infrastructure. Therefore, one of the major ethical concerns with NIPT is informed decision-making [130,131,132].

NIPT is a costly examination that is unavailable to numerous patients and is only reimbursed in selected countries, including Belgium, the Netherlands, and Germany. However, the continuously lowering costs, increasing diagnostic capabilities, and potential for use during the entirety of pregnancy are prospectively advantageous [102,133,134,135]. The significant enhancement in performance has resulted in the fast and extensive acceptance of NIPT, substantially altering the conventional framework of prenatal screening. A recent study described the current use of NIPT in Europe, Australia, and the USA (24 countries) and found considerable variation in the way in which NIPT is used worldwide, as well as between European countries. Countries with high NIPT uptake (>50%) showed corresponding decline in second-trimester serum screening [136]. Nevertheless, NIPT has drawbacks. Its diagnostic breadth is restricted. Indeed, it occasionally produces false positives. NIPT may yield false positives in cases of placental mosaicism, maternal neoplastic conditions (including rare autosomal trisomies), or in cases involving a deceased fetus, such as a vanishing twin [137,138,139]. In conclusion, NIPT is an advanced screening test, incurs significant expenses, is sometimes not reimbursed in many affluent nations, has a restricted diagnostic range, and necessitates confirmation of positive results by invasive procedures.

## 10. AFP Limitations, Future Research Directions, and Conclusion

Aberrant levels of AFP have been linked to a higher risk of congenital abnormalities, poor obstetric outcomes, MAP, and other serious conditions discussed in this review. MS-AFP values require careful interpretation due to the influence of various factors. A deeper understanding of the parameters influencing MS-AFP levels is essential for improving interpretation in prenatal screening and risk assessment. Notably, determining AFP concentrations has undergone significant advancements through advanced technologies that may help identify specific levels associated with certain diseases. These results highlight the value of AFP screening in the early stages of pregnancy to detect and treat high-risk pregnancies and enhance outcomes for both the mother and fetus.

AFP measurement has been utilized for four decades in prenatal screening. It is anticipated that MS-AFP measurement will continue to be utilized, though its role is evolving. In contemporary practice, AFP may function best not as a universal screening test but as a context-dependent tool: valuable in specific populations (high-risk and resource-limited settings) and when integrated with other assessments (ultrasound, NIPT, and clinical risk factors).

Comprehensive screening methods and effective treatment strategies require further research and development. The correlation between AFP and adverse outcomes is well documented. However, the critical gap is whether AFP-based screening translates into improved outcomes. Future research must prioritize this question: demonstrating clinical utility requires showing not only association with poor outcomes but also that screening-driven interventions reduce their occurrence. Integrating both AFP and ultrasound approaches judiciously, rather than relying on either in isolation, may provide the optimal balance of detection, resource utilization, and outcome improvement. The field would benefit from moving beyond debates about whether AFP “works” to pragmatic questions about for whom, in what contexts, and with what downstream actions AFP screening adds value. This evidence-based, context-specific approach represents the future of prenatal screening as technologies continue to evolve.

**Critical Distinction**: Biomarker vs. Pathogenic Agent. A fundamental unresolved question merits explicit discussion: does AFP serve merely as a biochemical marker reflecting underlying pathology, or does it actively contribute to the pathophysiological mechanisms causing fetal anomalies, obstetrical problems, and pregnancy complications? This distinction has profound implications for research priorities and clinical interpretation. The current evidence from this review suggests that AFP functions primarily as a marker rather than a causal agent. However, the known biological functions of AFP (carrier protein for fatty acids and bilirubin; potential immunomodulatory effects; possible involvement in growth regulation) raise the possibility that in some contexts, abnormal AFP levels could contribute to pathogenesis. Definitive resolution of this question requires: (1) animal knockout and overexpression studies examining whether AFP manipulation affects pregnancy outcomes; (2) proteomic analyses of AFP-binding partners in normal versus complicated pregnancies; and (3) mechanistic studies of AFP’s biological roles beyond its carrier function. This may lead to the discovery of novel potential functions of AFP and help in monitoring treatment protocols in the future.

**Clinical Takeaways: AFP in Pregnancy—Practical Guidance**. Regarding when to order MS-AFP, screening should be offered between 15–20 weeks of gestation, ideally as part of integrated or sequential screening (including nuchal translucency, hCG, uE3, and inhibin A); in resource-limited settings, MS-AFP retains strong standalone value for NTD risk detection. When interpreting an elevated MS-AFP (≥2.5 MoM), a Level II ultrasound should be initiated; if no structural anomaly is identified, evaluation for placental pathology and enhanced third-trimester surveillance are warranted, and very high values (≥5.0 MoM) in multiparous women warrant specific assessment for placenta accreta spectrum. A low MS-AFP (<0.7 MoM) should prompt evaluation for chromosomal aneuploidies, with genetic counselling and consideration of NIPT or invasive testing based on combined-risk assessment. AFP adds unique value beyond ultrasound in two key contexts: detection of closed or skin-covered spinal defects that are sonographically occult, and early biochemical signalling of placental dysfunction before structural anomalies become visible on imaging. Conversely, AFP does not change management when there is an isolated borderline elevation (2.0–2.5 MoM) alongside a normal anatomy ultrasound and no other risk factors; in centres with access to high-resolution ultrasound and NIPT, routine AFP screening for structural anomaly detection provides only incremental value. Finally, AFP and NIPT should be regarded as complementary rather than competitive tools: NIPT does not screen for NTDs or placental complications, and MS-AFP continues to provide unique diagnostic information in these domains.

## Figures and Tables

**Table 1:** Overview of key methods for AFP detection based on recent research.

AFP Detection Methods	Principal Use	Detection Limit	References
Fluorescent aptasensors	Förster resonance energy transfer (FRET)	400 pg/mL	Zhou, L. et al., 2019 [30]
Simultaneous Detection Methods	Catalytic hairpin assembly (CHA) amplification with quantum dots and N-methyl mesoporphyrin IX (NMM)	3 fg/mL	Chen, P. et al., 2022 [31]
Digital quantification	Microfluidic array chips incorporating modified magnetic microparticles (MMPs) and Poisson distribution analysis	1 fg/mL	Tian, S. et al., 2018&2019 [32,33]
Electrochemical aptasensors	Nanocomposites graphene oxide-based	3 pg/mL	Yang, S., Zhang, F., Wang, Z. & Liang, Q., 2018 [34]
Surface-enhanced Raman spectroscopy (SERS)	Combine DNA hydrogels with Raman tags	50 pg/mL	Wang, Q. et al., 2020 [35] Ma, H. et al., 2017 [36]
Photoelectrochemical (PEC) biosensors	Light to generate an electrical signal	0.01 ng/mL	Li, X., Pan, X., Lu, J., Zhou, Y. & Gong, J., 2020 [37] Xu, R. et al., 2015 [38]
Microchip-based enzyme-linked immunosorbent assay (ELISA)	Enzyme-linked immunosorbent assay (ELISA)	1 pg/mL	Liu, Y. et al., 2009 [39]
Enhanced fluorescence ELISA (FELISA)	Human alpha-thrombin to trigger fluorescence “turn-on” signals	10–8 ng/mL	Wu, Y. et al., 2017 [40]
Colorimetric	Gold nanoparticles act as colorimetric agents, then a smartphone app captures the color changes and calculates the AFP concentration in the sample	0.083 ng/mL	Liu, J., Geng, Q. & Geng, Z., 2024 [41]

## Data Availability

No new data were created or analyzed in this study. Data sharing is not applicable to this article.

## Abbreviations

ACOG	American College of Obstetricians and Gynecologists
AFP	Alpha-fetoprotein
ALB	Albumin
cffDNA	cell-free fetal DNA
CHA	catalytic hairpin assembly
CHD	congenital heart defects
CLIA	chemiluminescent immunoassay
ELISA	enzyme-linked immunosorbent assay
FELISA	enhanced fluorescence enzyme-linked immunosorbent assay
FRET	Förster resonance energy transfer
GCTs	germ cell tumors
HB	hepatoblastoma
HCC	hepatocellular carcinoma
hCG	human chorionic gonadotrophin
IUGR	intrauterine growth restriction
MAP	morbidly adherent placenta
MMPs	modified magnetic microparticles
MoM	multiples of the median
MS-AFP	maternal serum alpha-fetoprotein
NIPT	non-invasive prenatal testing
NMM	N-methyl mesoporphyrin IX
NTDs	neural tube defects
PAPP-A	pregnancy-associated plasma protein A
PAS	pMAPlacenta accreta spectrum
PEC	photoelectrochemical
PP	placenta previa
QDs	quantum dots
SERS	surface-enhanced Raman spectroscopy
SIDS	sudden infant death syndrome
uE3	unconjugated estriol
